# A materials terminology knowledge graph automatically constructed from text corpus

**DOI:** 10.1038/s41597-024-03448-0

**Published:** 2024-06-07

**Authors:** Yuwei Zhang, Fangyi Chen, Zeyi Liu, Yunzhuo Ju, Dongliang Cui, Jinyi Zhu, Xue Jiang, Xi Guo, Jie He, Lei Zhang, Xiaotong Zhang, Yanjing Su

**Affiliations:** 1 School of Computer and Communication Engineering, University of Science and Technology Beijing, Beijing, 100083 China; 2 Beijing Advanced Innovation Center for Materials Genome Engineering, Institute for Advanced Materials and Technology, University of Science and Technology Beijing, Beijing, 100083 China; 3 Liaoning Academy of Materials, Shenyang, 110000 Liaoning China; 4 Shunde Innovation School, University of Science and Technology Beijing, Guangdong, 528399 China; 5 Beijing Key Laboratory of Knowledge Engineering for Materials, Beijing, 100083 China

**Keywords:** Computational science, Theory and computation

## Abstract

A scalable, reusable, and broad-coverage unified material knowledge representation shows its importance and will bring great benefits to data sharing among materials communities. A knowledge graph (KG) for materials terminology, which is a formal collection of term entities and relationships, is conceptually important to achieve this goal. In this work, we propose a KG for materials terminology, named Materials Genome Engineering Database Knowledge Graph (MGED-KG), which is automatically constructed from text corpus via natural language processing. MGED-KG is the most comprehensive KG for materials terminology in both Chinese and English languages, consisting of 8,660 terms and their explanations. It encompasses 11 principal categories, such as Metals, Composites, Nanomaterials, each with two or three levels of subcategories, resulting in a total of 235 distinct category labels. For further application, a knowledge web system based on MGED-KG is developed and shows its great power in improving data sharing efficiency from the aspects of query expansion, term, and data recommendation.

## Introduction

Machine learning (ML) and artificial intelligence (AI) have demonstrated the power in speeding up new materials discovery in a cost-efficient manner^1–9^. Materials science data, as the foundation of ML-based and AI-based materials discovery applications, has become a critical premise, and plenty of materials data infrastructures have been established to collect, host, provide and analyze materials data to stakeholders^7,10^. The increasing capabilities of computational science in general have accelerated infrastructures becoming materials discovery platforms such as Novel Materials Discovery (NOMAD)^11^, Automatic FLOW for Materials Discovery (AFLOW)^12^, the Materials Project^13^, and Materials Cloud^14^. Open Databases Integration for Materials Design (OPTIMADE)^15^ provides a universal application programming interface (API) to make these heterogeneous platforms accessible and interoperable. There is also a massive surge in autonomous experiments and literature data, and the concerns about long-term storage, integration and exchange over multi-source materials data are emerging like Materials Data Facility (MDF)^16^ and MGEDATA^17^. Most of the state-of-art materials data infrastructures concentrate on specific materials classification schemes by their functional properties (mechanical, optical, electronic), topological characteristics (surface, bulk, nanoparticles), or by materials types (steel, ceramics, composites). The alignment and fusion of material heterogeneous data across infrastructures is difficult. Therefore, a scalable, reusable, and broad-coverage unified material knowledge representation shows its importance and will bring great benefits to data sharing among materials communities.

A knowledge graph (KG) for materials terminology, which is a formal collection of term entities and relationships, is conceptually important to achieve this goal. It is essential for enhancing the readability and maintainability of the materials data infrastructure, which provides a standardized naming approach for materials data, unifying the vocabulary with different expressions into a single term to improve data consistency and facilitates semantic reasoning and complex searches^18^. Furthermore, the KG for materials terminology maintains the materials terms into a concept network, making it easier for researchers to share knowledge and incorporate the existing knowledge into their current research. However, creating a well-knowledgeable KG for materials terminology in material science is a daunting task. A KG schema, which is usually represented by an ontology, must be defined, including suitable formats and standards for encoding^19,20^. Currently, there are diverse ontologies and informal standards being utilized in materials science. NOMAD Meta-info^21^, Experimental Structure-Property Correlation Database Framework (ESCDF)^22^, and Open Knowledgebase of Interatomic Models (OpenKIM)^23^ are the first attempts to categorize computational results in atomistic materials science. Plinius ontology^24^ is the earliest materials ontology which is developed for ceramic materials that covers the concept of the chemical composition of materials. ONTORULE steel ontology is developed for the steel industry which aims to build the conceptual model with the steel use case^25^. Semantic Laminated Composites Knowledge Management System (SLACKS) ontology is developed for the engineering of laminated composites structures which integrates relevant domains of the product life cycle^26^. Battery Interface Ontology (BattINFO) is a foundational resource for harmonizing battery knowledge representation and enhancing data interoperability (https://github.com/BIG-MAP/BattINFO). The Elementary Multiperspective Material Ontology (EMMO) aims at the development of a standard representational ontology framework based on current materials modelling and characterization knowledge (https://github.com/emmo-repo/EMMO)^27^. MatOnto, a machine-processable ontology, is used to integrate data across disparate materials databases^28^. Physical Information File (PIF) provides a hierarchical data structure for storing materials data^29^. Materials Ontology contains several sub-ontologies corresponding to substance, process, environment, and property, offering a standard data exchange format between heterogeneous databases^30^. MatOWL, short for Materials Ontology Web Language, is automatically constructed from available materials schemas and can be mapped to other materials ontologies using logical rules^31^. The vast majority of these ontologies are artificially created by material scientists in various sub-fields, with different structures and standards. Fitting existing and novel data into such an ontological framework would still be a tedious, human-intensive, and error-prone task for humans.

Due to the rapid development of AI techniques, the construction of a material terminology KG has entered a new era in the past few years, with a focus on automation. The open-source Python framework, Propnet^32^, expands the existing materials property dataset by systematizing and connecting material properties. The Materials Experiment Knowledge Graph (MekG)^33^ showcases the complete hierarchical relationships of material experiments in the form of a graph database. NanoMine^34^ leverages the Whyis knowledge graph framework and integrates diverse data from over 1,700 polymer nanocomposite experiments. Materials Design Ontology^35^ establishes an information bridge among different data providers in computational materials science through its ontological framework, facilitating interoperability among computational materials databases.

By using semi-automatic and automatic extraction methods based on natural language processing (NLP) techniques, domain experts can rapidly and in bulk extract terms and vocabulary from natural language text corpus, significantly reducing the difficulty of KG construction^36–39^. Named entity recognition (NER) and relation extraction tasks are considered as key components of this process. Recently, pipelines for automatic data extraction of organic^40,41^, inorganic chemical substances^37,38,42^ and alloys^39,43^ from articles in the fields of chemistry and materials science have been introduced using NLP techniques. With the advancement of large language models (LLMs), MatKG^44^ is automatically generated, forming a comprehensive knowledge graph spanning traditional material-structure-property-processing paradigms. These developments represent significant contributions to the ongoing evolution of materials science. In our previous work, we proposed an automatic NLP pipeline for global analysis of data extracted from 14425 journal articles, capturing chemical compositions and property data of superalloys^39,45,46^. AI tools and techniques can be used to simplify and automate the establishment of a well-knowledgeable terminology KG.

In this work, we propose a KG for materials terminology, named Materials Genome Engineering Database Knowledge Graph (MGED-KG), which is automatically constructed from text corpus by NLP. MGED-KG is the most comprehensive KG for materials terminology in both Chinese and English languages with the amount of 8,660 terms and their explanations, covering 11 principal categories of fundamentals of materials science, metals, inorganic non-metallic materials, organic polymer materials, composites, information materials, biomedical materials, energy materials, functional materials, natural materials and their products, and nanomaterials. Each of the principal category encompasses two or three levels of subcategories, resulting in a total of 235 distinct category labels. We design an NLP-based automatic construction method of material terminology from Comprehensive Dictionary of Materials (Second Edition)^47^ text corpus, starting from corpus preprocessing, NER of term entities, relation construction among entities and finally forming MGED-KG.

MGED-KG plays an important role in improving data sharing efficiency from the aspects of query expansion, term, and data recommendation. In terms of query expansion, MGED-KG is used to complete and standardize user input automatically. For term recommendation, MGED-KG can help users to find more related terms according to their focuses and explore more latent knowledge. To demonstrate the capability of MGED-KG in data recommendation, we integrate it to our National Materials Data Management and Service platform (NMDMS). NMDMS^48^ is a material database we developed in the early stages for the dissemination and sharing of heterogeneous materials science data. There are 12,251,040 pieces of data published in NMDMS since 2018, under 87 categories and 1,912 user-defined schemas from 45 projects. To leverage the term relationships, MGED-KG is embedded into NMDMS platform to construct the correlation between data instances, which helps realize the data instance recommendation based on their semantic similarities during data retrieval and accelerates the valuable data discovery efficiently. We develop an ontology using RDF-based semantics for MGED-KG, thereby facilitating cross-domain interoperability and comprehension. Our work lays an overall foundation to build a unified and coherent material semantic KG for the underlying basic terminologies in different material subfields. It powers great potential to accelerate materials data sharing and integration in a wider range to facilitate data-driven materials research.

## Result

### Materials terminology extraction by text mining

**Fig. 1:**
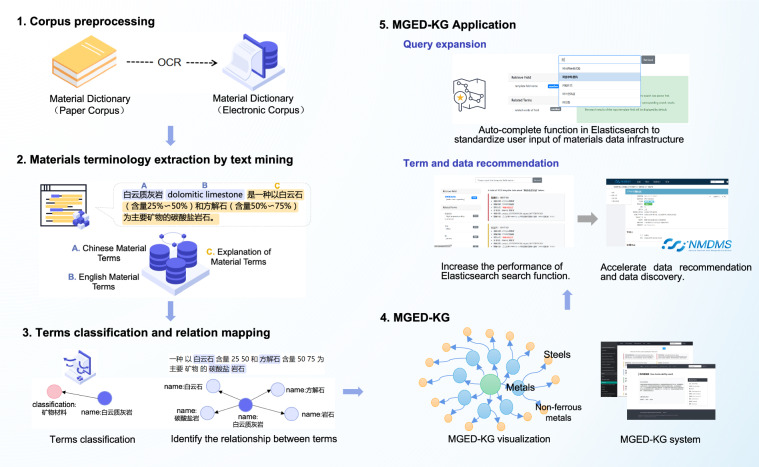
The semantic workflow of materials terminology extraction, construction of MGED-KG and its application.

The semantic workflow of materials terminology extraction, construction of MGED-KG and its application is shown in Fig. 1. The whole workflow starts from corpus preprocessing, materials terminology extraction by text mining, terms classification and relation mapping, followed by forming a MGED-KG web system. MGED-KG web system contains three application scenarios by providing query expansion function, term and data recommendation coupled with materials database.

We selected the classic paper corpus of the Comprehensive Dictionary of Materials (Second Edition) as the original Chinese text corpus. This dictionary is renowned as the world’s first comprehensive dictionary of materials science, authored by Academician Changxu Shi as the honorary editor, Chief Editor Boyun Huang, and over 500 experts and scholars. The raw paper corpus was scanned and transferred into electronic corpus by optical character recognition (OCR) technique to make it readable by computer. Subsequently, text preprocessing was carried out to ensure the quality of the corpus. This included converting full-width characters to half-width characters, converting uppercase to lowercase, filtering out line breaks, headers, and footers on each page, followed by manual proofreading and correction.

For terminology extraction, a rule-based NER method was utilized after paragraphs segmentation. For each paragraph, it consists of a Chinese material term, its English version, and its explanation. Regular expression was tailored for terms and their explanations recognition. Totally, a dataset of Chinese-English material terminology, containing 8,660 terms and their explanations, was obtained and the precision of NER achieved 98.74% by manual check.

**Fig. 2:**
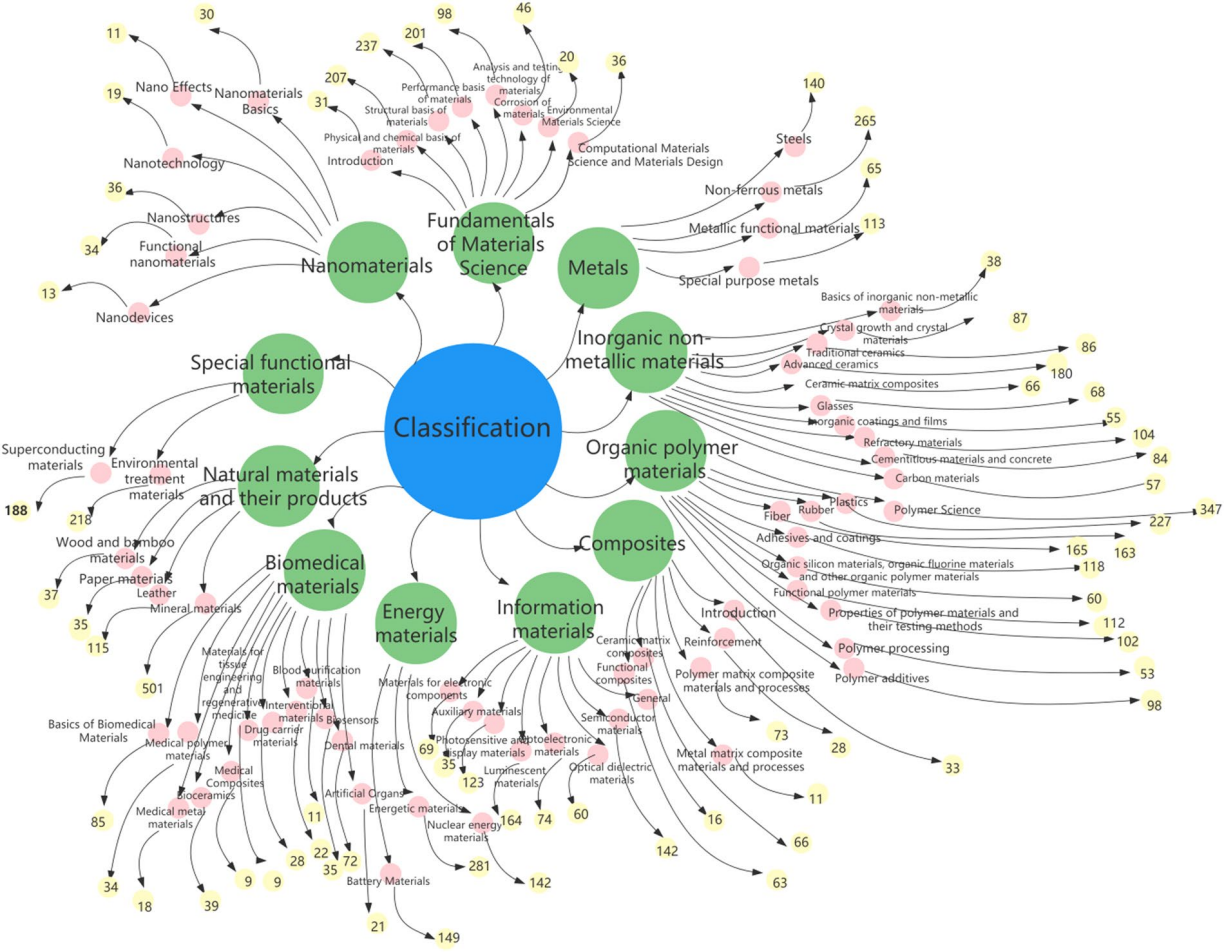
The visualization of MGED-KG in hierarchical structure with first two levels of categories and the number of third level categories below them.

To further construct the structure of MGED-KG, terms classification was first achieved. Materials category labels were assigned to each term entity determined by the catalog of Comprehensive Dictionary of Materials (Second Edition). The terminology classification is hierarchical in three levels, with a total of 235 category labels. The first level comprises fundamentals of materials science, metals, inorganic non-metallic materials, organic polymer materials, composites, information materials, energy materials, biomedical materials, natural materials and their products, functional materials, as well as nanomaterials. And for the second and third level, the term categories are more nuanced. For example, the term “ferrovanadium” has the category label of “metals ->steels -> iron”. Figure 2 shows the hierarchical classification structure of MGED-KG with first two levels of categories and the number of third level categories below them. The hierarchical classification of terms will further act as the ontology of MGED-KG. Next, relationships between terms are automatically captured, that is, if other terms appear in the explanatory text of a term, then the term is automatically associated with other terms. Therefore, the term linkage at the knowledge level is established, which can be used as the MGED-KG entity relation.

### MGED-KG visualization

**Fig. 3:**
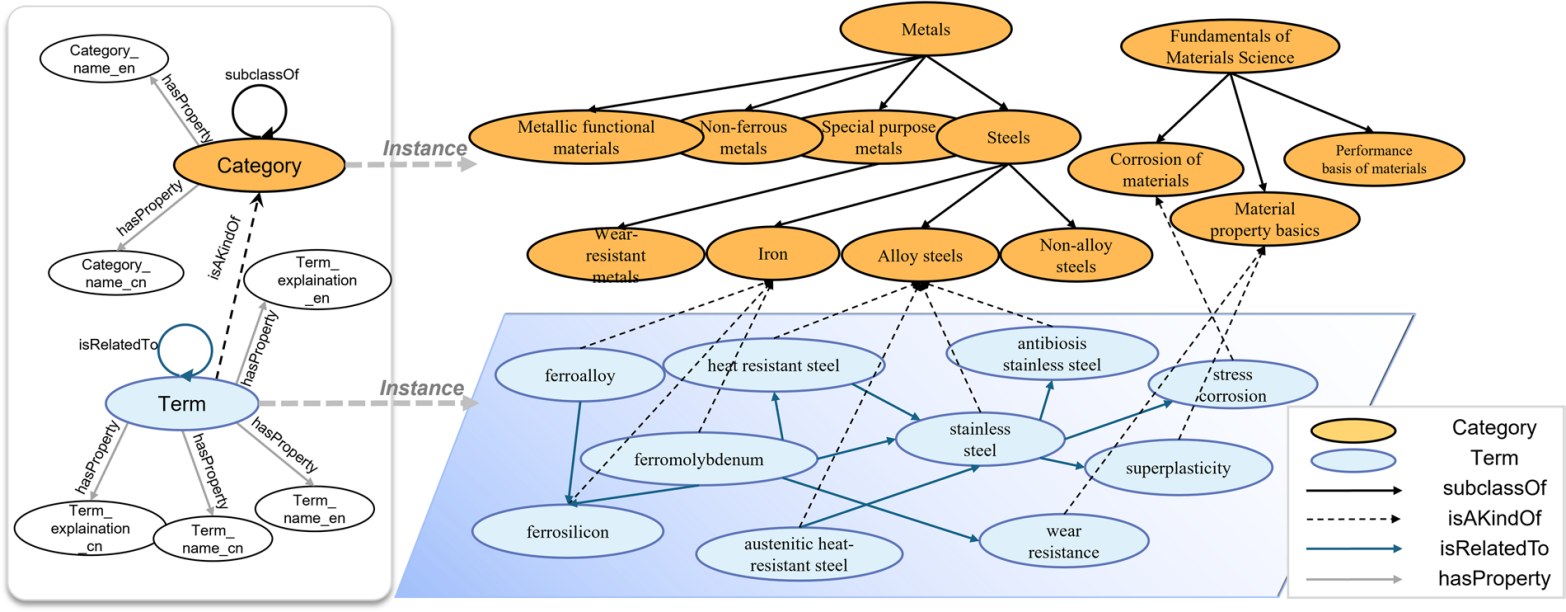
MGED-KG Material Knowledge Graph Structure Diagram.

MGED-KG encompasses not only the classification and explanation of material terms but also the semantic relationships and interactions between terms. Figure 3 provides an overview of the structure of MGED-KG. The left part shows the schema of ontology, and right part illustrates some instances related to the terms and categories. The “Category” class represents high-level conceptual classifications within the field of materials science, such as “Metals”, “Fundamentals of Materials Science”, “Steels”, “Non-ferrous metals”, and “Material property basics”. Each “Category” can be further refined into subcategories, with the “subclassOf” relationship expressing the hierarchical structure between classes. For example, “Steels” can be further classified with “Non-alloy steels”, “Iron”, “Alloy steels”, etc. Within “Category”, the “hasProperty” relationship points to the category’s English name and Chinese name. The “Term” class represents the specialized terminology of materials science, describing attributes such as the types, structures, methods, applications, or properties of materials. The “hasProperty” relationship for “Terms” points to the term’s English name, Chinese name, English explanation, and Chinese explanation. Each “Term” is assigned to a specific term “Category”, with the “isAKindOf” relationship establishing a clear belonging relationship. For instance, “ferroalloy” is a kind of “Iron”, and “superplasticity” is a kind of “Material property basics”. The “isRelatedTo” relationship between “Term” entities reveals the semantic connections between terms, enhancing the understanding of complex interactions among entities within the field of materials science.

### MGED-KG system

**Fig. 4:**
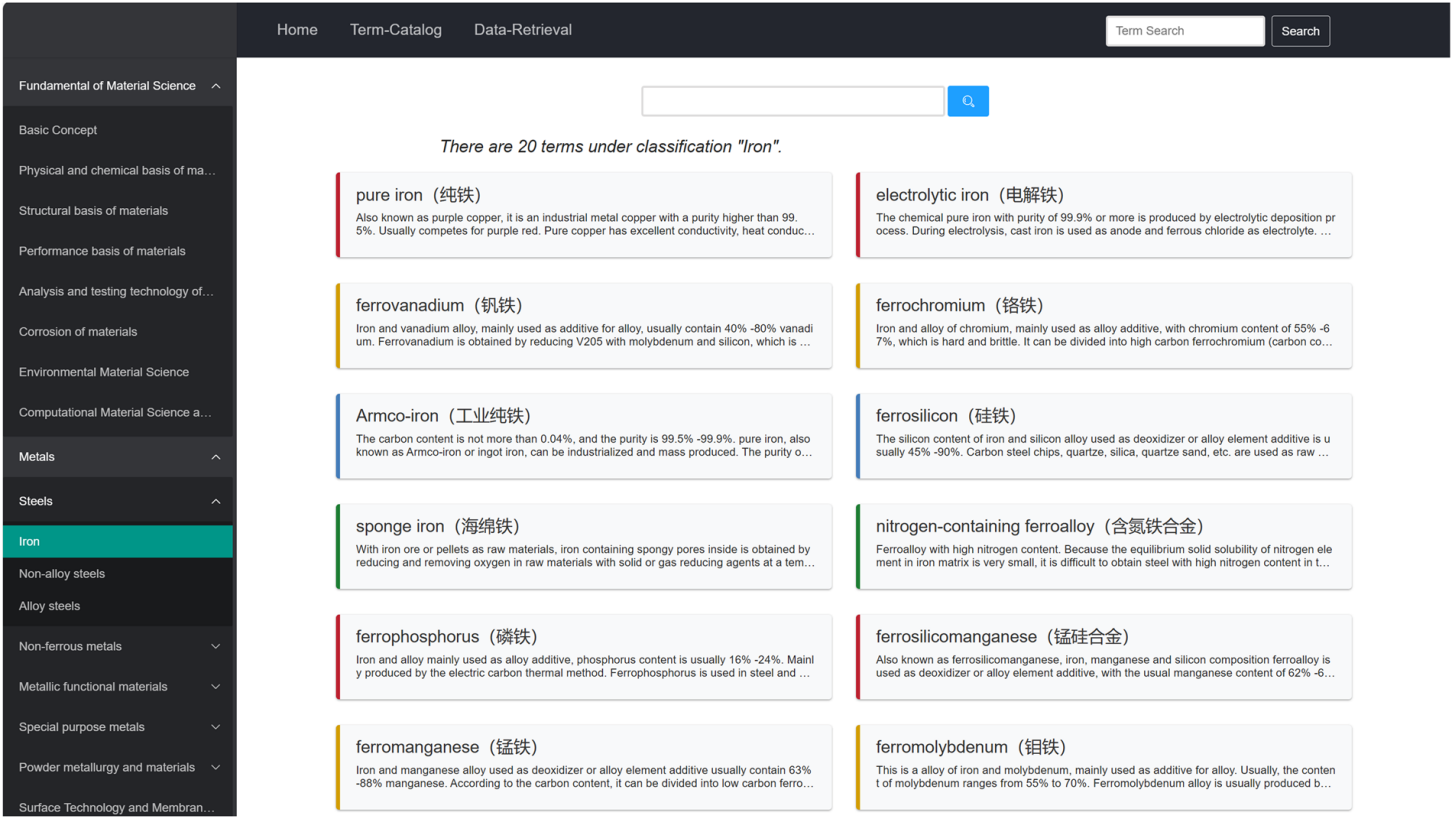
An example of term catalog. The left section of the page is the three-level hierarchical categories, and the right section shows there are 20 material terms under the classification of metal material “Iron”.

**Fig. 5:**
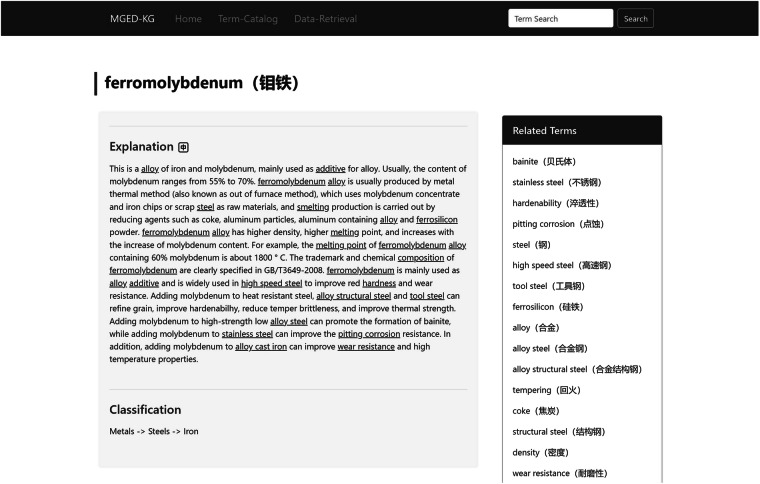
An example of a term page, containing the Chinese and English name (in parentheses) of the term, the explanation, and the classification. In the right section, there are related terms that associate with current term according to the term relation in MGED-KG. A language switch button for “Explanation” is provided, offering term explanations in Chinese and English. Additionally, clicking on terms with underlines in the explanation allows users to directly navigate to the detailed page for that specific term.

With the support of web technologies, MGED-KG can be distributed in a digital form through online platform. We used the Django framework (https://www.djangoproject.com/), which is a highly scalable Python web framework that can quickly develop and deploy complex web applications, to construct a MGED-KG system(http://mged.nmdms.ustb.edu.cn/MGEDKG/) for data standardization and terminology sharing among materials community. The MGED-KG system includes functional modules of term catalog, term retrieval, term recommendation and data recommendation associated with NMDMS database. The term catalog function provides navigation of 8,660 terms organized by three-level hierarchical structure. The term retrieval function provides a gateway for discovering materials terms and their explanations. Figure 4 shows the result list filtered by term catalog navigation, and Fig. 5 provides an example of a term page.

## Discussion

**Fig. 6:**
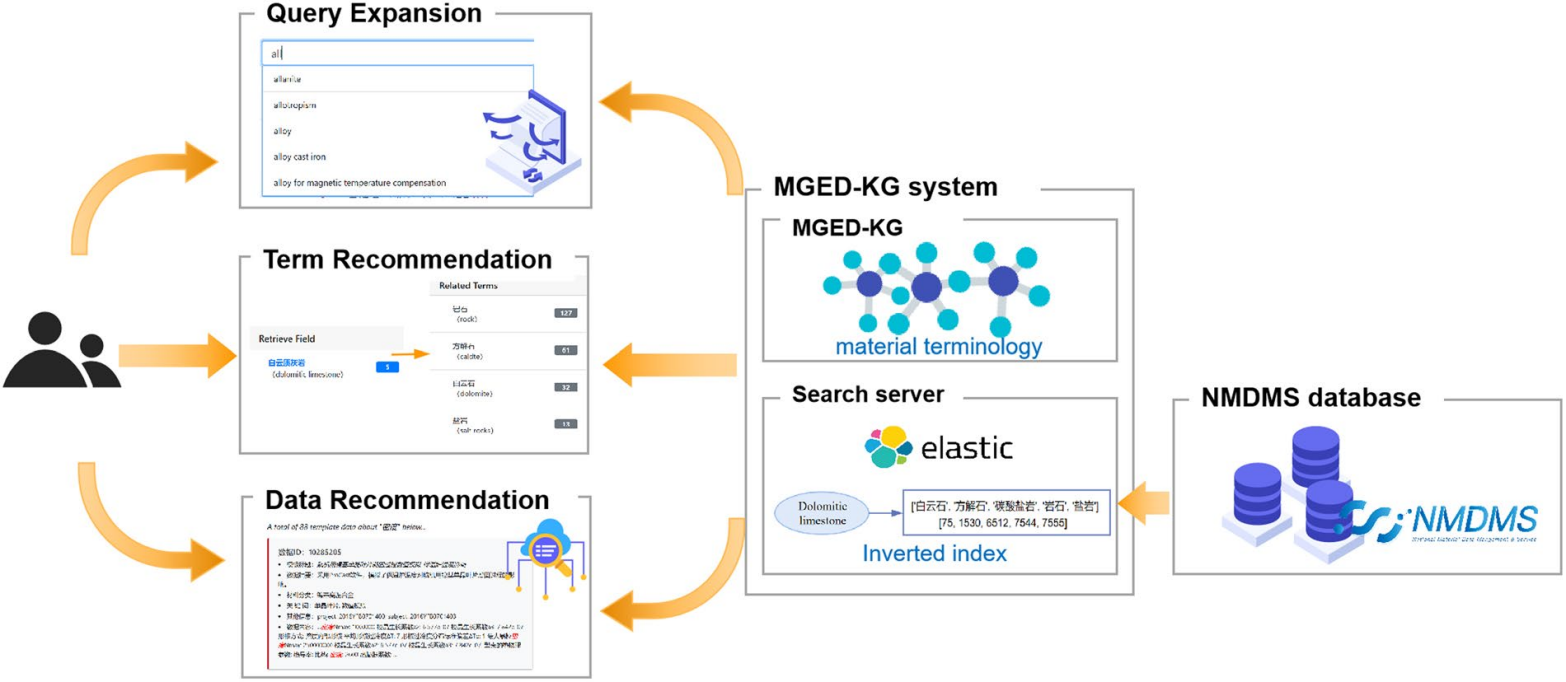
MGED-KG applications from query expansion, term, and data recommendation aspects.

Due to the benefits of the knowledge graph, MGED-KG can effectively reduce the difficulty of materials terminology reuse in a semantic knowledge repository and enhance the possibility of effective collaboration. This allows engineers, researchers, and other professionals in different material domains to quickly transform knowledge into required format and aid in the utilization of knowledge within intelligent systems. To demonstrate its capability, MGED-KG was successfully applied to three scenarios regarding query expansion, term, and data recommendation, as shown in Fig. 6.

### Query expansion

**Fig. 7:**
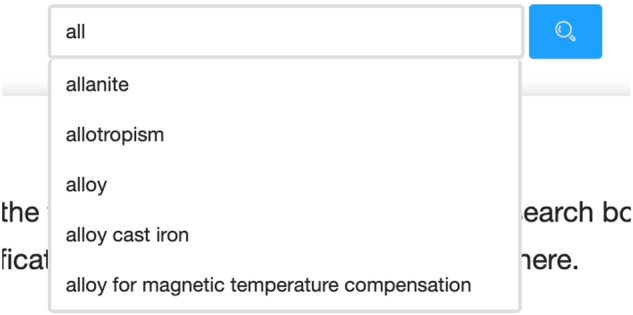
Query expansion function for user input completion, standardization, and data retrieval in MGED-KG system.

MGED-KG can be used to complete and standardize user input automatically, and significantly improve the user experience when retrieving material terms. It automatically recommends potential terms related to a user’s input which may be only a part of a term. For example, when a user enters “all” as a query term, the system automatically suggests possible query expansion terms such as “allanite”, “allotropism”, “alloy”, “alloy cast iron”, and “alloy for magnetic temperature compensation”. Figure 7 shows the query expansion function for user input completion, standardization, and data retrieval in MGED-KG system.

Elasticsearch (ES) (https://www.elastic.co/cn/elasticsearch/) is a distributed search engine based on inverted indexes which helps to build an index of material terms for autocompletion for query expansion. ES provides the completion suggester feature, which leverages inverted indexes to construct a specialized data structure called a completion index. ES comes with various built-in analyzers, but these analyzers are not particularly suitable for materials terminology corpus, as they do not segment text according to materials terminology conventions. The Lucene IK analyzer, on the other hand, is a standard Chinese text analyzer. It segments text based on a defined dictionary for a field and supports user-configurable custom dictionaries. Therefore, we have used the previously extracted material terms as a custom lexicon. We integrated the Lucene IK analyzer into ES using the IK Analysis plugin to achieve improved segmentation results. In this way, the precision, recall and F1 score of word segmentation for material terms has been successfully increased by 18.72%, 8.91% and 14.15%, respectively. When documents are indexed, ES extracts partial or complete text from specified fields and adds them to the completion index. When a user enters a query, ES sends a completion suggestion query request to search for relevant completion suggestions in the completion index. ES returns a list of completion results related to the user’s input, including matching phrases or suggested autocompletion terms, based on the query content and settings. The query expansion functionality enables the system to quickly respond and provide relevant autocompletion suggestions during the user input process, thereby enhancing search experience and query precision.

### Term and data recommendation

**Fig. 8:**
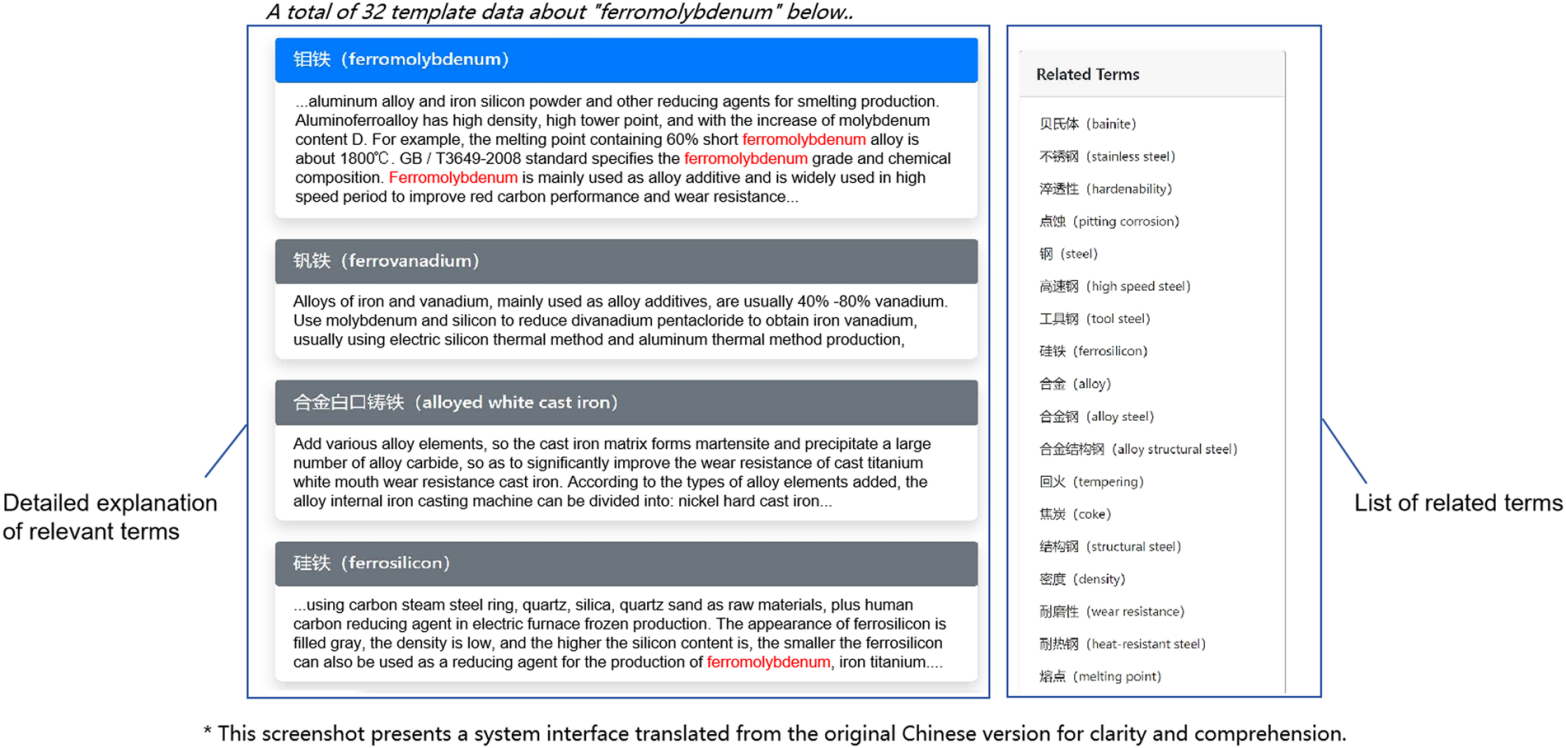
Term recommendation by MGED-KG.

The terminology recommendation feature aims to enhance information retrieval and knowledge acquisition in the materials domain. This feature leverages the MGED-KG, which we have constructed, to automatically recommend a list of related terms. For example, as shown in Fig. 8, when a user enters a term for retrieval, such as “ferromolybdenum”, our system automatically provides a list of terms related to “ferromolybdenum”, including “bainite”, “stainless steel”, “hardenability”, “pitting corrosion”, “steel”, “high-speed steel”, “tool steel”, “ferrosilicon”, “alloy”, “alloy steel”, “alloy structural steel”, “tempering”, “coke”, “structural steel”, “heat resistant steel”, and “cast iron”. During terminology recommendation, we utilized the connections and associations within the knowledge graph to infer terms relevant quickly and accurately to the user’s input. Through this approach, our system can provide a more comprehensive and precise list of terms, aiding users in better understanding and accessing knowledge in the materials domain.

Furthermore, MGED-KG has been embedded into NMDMS platform to construct the correlation between data instances, which help realize the data recommendation during data retrieval and accelerate the valuable data discovery efficiently. For example, when the keyword “single crystal” in English or “单晶” in Chinese is retrieved in NMDMS platform, there are totally 124 data instances hit by the retrieval. At the same time, MGED-KG system automatically returns a term list containing “single crystal” and then send these related terms to NMDMS platform again for related data instances retrieval.

**Fig. 9:**
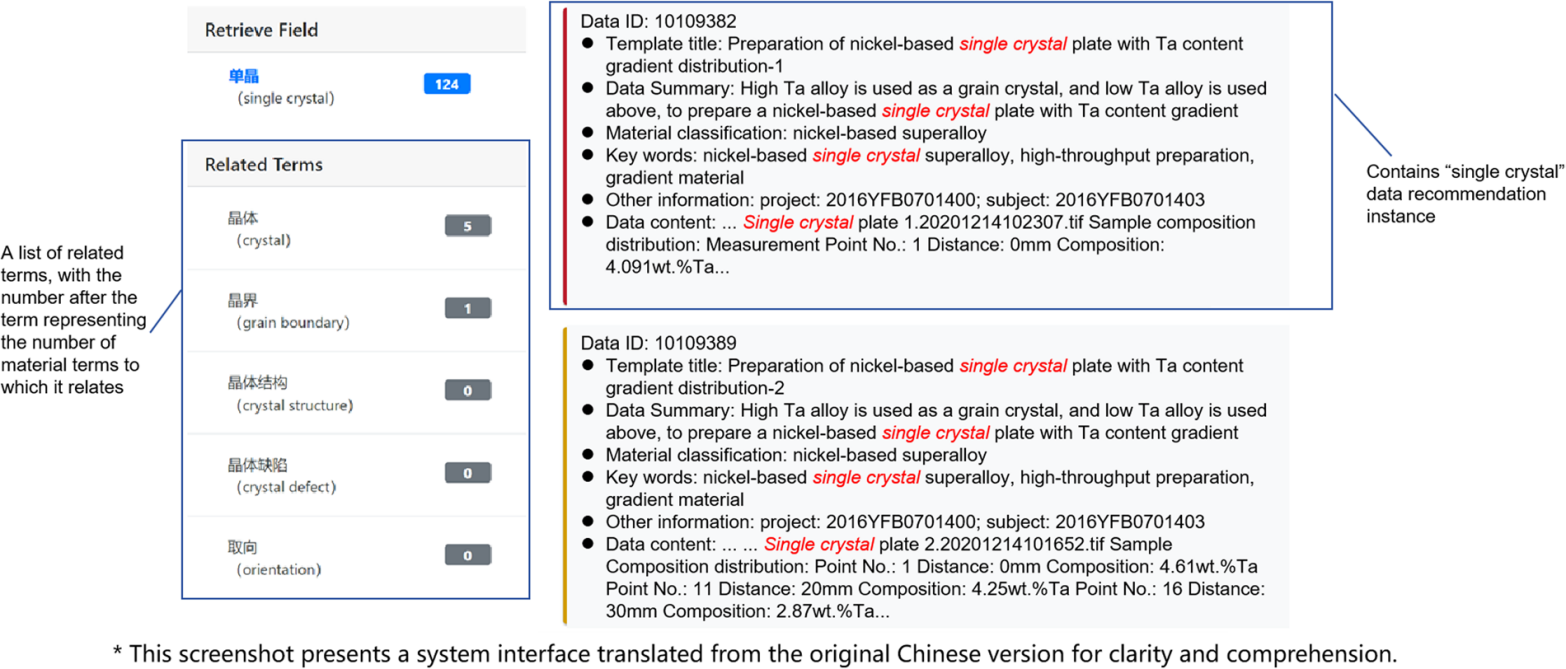
An example of data retrieval in NMDMS platform with data recommendation.

The implementation of the data recommendation functionality is also based on ES engine. We have constructed a specialized ES index in NMDMS platform dedicated to the materials domain, which contains a large volume of materials data and their related properties. When a user enters a keyword, we utilize the search capability of ES to quickly retrieve data matching the keyword from the index. Through fast search and interactive access to data details, users can conveniently access the required materials data, effectively supporting their research and decision-making processes. Figure 9 shows an example of data retrieval in NMDMS and in the result there are recommended data instances by related terms in the left section the of web page.

## Methods

### Corpus acquisition and preprocessing

In our research, we chose the paper book of the Comprehensive Dictionary of Materials (Second Edition) as the original text corpus. We obtained the electronic version by scanning it. Subsequently, we processed the images in the electronic version in batches and extracted the text content from them. Extracting text from images requires two steps: image processing and OCR. In the first step, we preprocessed the images to remove noise and unnecessary information and improve the recognition rate of OCR. Then, we used OCR technology to recognize the text information in the images and convert it into editable text format. OCR recognition technology can convert characters in images into computer-readable character codes. However, due to the existence of some special characters and non-standard text in the original text corpus, the precision of OCR recognition is not high enough, and further preprocessing was done. This process includes the conversion of special characters. For example, fullwidth to halfwidth, uppercase to lowercase, unity between numbers, letters and Chinese, and filter and process information such as line breaks, header, and footer in the text to get more orderly text data. For some complex images, OCR technology may not be able to correctly recognize special characters, so we manually corrected them and checked for grammar and spelling errors to ensure recognition precision.

### NER of terms

This study utilized a set of regular expressions to achieve NER of terms in the Comprehensive Dictionary of Materials (Second Edition) corpus. A primary regular expression consisted of combinations of Chinese characters and alphanumeric characters. To handle some special cases, we also used four additional regular expressions that consider special symbols such as plus and minus signs, degree symbols, Greek letter gamma. There were specific expressions for matching terms that include Roman numerals in their Chinese parts and for matching terms that do not contain English. Additionally, the explanations for the terms started with the character “见” (meaning “see”). By applying this set of regular expressions, we accurately extracted the target terms and their corresponding explanations effectively. The accuracies of each kind of terms are listed in Table 1 and the total average of NER precision reaches 98.74%.

**Table 1:** The NER rules and precision of material terms.

No.	Regular expression	Regular expression application	Examples (Chinese Term - English Term)	Number of terms hit	Terms in total	Precision
1	^([-A-Za-z0-9]*[-\u4e00-\u9fa5: /]+)[\s]*([-A-Za-z]+[-\s“A-Za-z0-9;；:()/]+[A-Za-z0-9]+)	Matching combinations of Chinese and alphanumeric characters	X射线荧光谱 X-ray fluorescence spectroscopy	7371	8660	85.12%
2	^([-\u4e00-\u9fa5,()/A-Za-z0-9±°γ]+)[\s]+([-\sA-Za-z0-9,;()/δ±°γ]+[A-Za-z0-9()]+)	Matching special symbols	1,1-二氨基-2,2-二硝基乙烯 FOX-7	74		0.85%
3	^([\u4e00-\u9fa5]+): ((([\u4e00-\u9fa5]+)[((](I|II|III)[))]), (([\u4e00-\u9fa5]+)[((](I|II|III)[))]))[\s]*([-A-Za-z]+[-\s“A-Za-z0-9;；:()/]+[A-Za-z0-9]+)	Matching cases where the Chinese part of the term includes Roman numerals	正磷酸锶锌: 锡 (II),锰 (II) strontium zinc orthophosphate activated by tin and manganese	26		0.30%
4	^([\u4e00-\u9fa5]+): (([\u4e00-\u9fa5]+)[((](I|II|III)[))])[\s]*([-A-Za-z]+[-\s“A-Za-z0-9;；:()/]+[A-Za-z0-9]+)	Matching cases where the Chinese part of the term does not include Roman numerals	硫化锌: 锰 (I) zinc sulfide activated by manganese	60		0.69%
5	^([-\u4e00-\u9fa5,0-9A-Za-zX]+)[\s]*见	Matching terms that do not include English and the explanation of the term starts with “见”	白焊合金 见银焊合金 (861页)	1020		11.78%
Total				**8551**		**98.74%**

### The comparison of word segmentation in Elasticsearch

We tested the precision of word segmentation before and after adding MGED-KG into ES for materials text. We selected a certain number of term explanations from the corpus as the dataset, imported the dataset into the ES database and created the corresponding index. To facilitate comparison, two indexes were created at the same time, one did not use MGED-KG and the other used. For indexes without MGED-KG, we used the default ES word divider for word segmentation. For indexes with MGED-KG, we added the custom material term dictionary to ES and use the custom word divider for word segmentation. In order to ensure the reliability of the experiment, multiple different datasets were used for the experiment, and the experiment was repeated many times to obtain more stable results. At the same time, datasets were selected to cover different classification of material terms to obtain more comprehensive experimental results.

To compare the segmentation effects of the two indexes, Precision, recall, and F1 score based on the confusion matrix were used as the metrics. Precision refers to the proportion between the number of correct material terms in the word segmentation results and the total number of word segmentation results, and recall rate refers to the proportion between the number of correct material terms in the word segmentation results and the number of all marked material terms. The F1 score, which weights precision and recall equally, as calculated from Eq. (1), can be used to measure the harmonic average of precision and recall.

1 $${\rm{F1}}\,{\rm{score}}=\frac{2\ast {\rm{Precision}}\ast {\rm{Recall}}}{{\rm{Precision}}+{\rm{Recall}}}$$

Table 2 shows the details of precision, recall and F1 score before and after using MGED-KG in ES. The precision, recall and F1 score of word segmentation for material terms has been successfully increased by 18.72%, 8.91% and 14.15%, respectively, after adding MGED-KG into ES.

**Table 2:** The precision, recall and F1 score before and after using MGED-KG in Elasticsearch.

	Precision (Without MGED-KG)	Precision (With MGED-KG)	Recall (Without MGED-KG)	Recall (With MGED-KG)	F1 score (Without MGED-KG)	F1 score (With MGED-KG)
Average	63.26%	72.45%	77.65%	83.30%	0.69	0.77
Increased	14.53%		7.28%		11.59%	

## Data Availability

MGED-KG are available at http://mged.nmdms.ustb.edu.cn/MGEDKG/. The corpus and ontology repository are accessible at Zenodo^49^.

## Code availability

The MGED-KG project is available at Zenodo^49^ https://zenodo.org/records/11315713.

This code is written in Python language, uses Django, Elasticsearch and other open-source libraries, and is compatible with a variety of operating systems, such as Windows, Linux. We provide detailed deployment steps and installation documentation so that users can quickly set up an environment and run code. We also maintain and update the code regularly to ensure its stability and availability.
